# Successful fishing for nucleus pulposus progenitor cells of the intervertebral disc across species

**DOI:** 10.1002/jsp2.1018

**Published:** 2018-06-27

**Authors:** Daisuke Sakai, Jordy Schol, Frances C. Bach, Adel Tekari, Nobuho Sagawa, Yoshihiko Nakamura, Samantha C.W. Chan, Tomoko Nakai, Laura B. Creemers, Daniela A. Frauchiger, Rahel D. May, Sibylle Grad, Masahiko Watanabe, Marianna A. Tryfonidou, Benjamin Gantenbein

**Affiliations:** ^1^ Department for Orthopaedic Surgery Tokai University School of Medicine Isehara Japan; ^2^ Center for Regenerative Medicine Tokai University School of Medicine Isehara Japan; ^3^ Department of Clinical Sciences of Companion Animals, Faculty of Veterinary Medicine Utrecht University Utrecht The Netherlands; ^4^ Tissue and Organ Mechanobiology, Institute for Surgical Technology and Biomechanics, Medical Faculty University of Bern Bern Switzerland; ^5^ Laboratory of Molecular and Cellular Screening Processes Centre of Biotechnology of Sfax, University of Sfax Sfax Tunisia; ^6^ Department of Orthopaedic Surgery University Medical Centre Utrecht Utrecht The Netherlands; ^7^ AO Spine Research Network, AO Spine International Davos Switzerland; ^8^ Department of Musculoskeletal Regeneration, AO Research Institute Davos Switzerland

**Keywords:** biologic therapies, culture systems, stem cell, tissue‐specific progenitor cells

## Abstract

**Background:**

Recently, Tie2/TEK receptor tyrosine kinase (Tie2 or syn. angiopoietin‐1 receptor) positive nucleus pulposus progenitor cells were detected in human, cattle, and mouse. These cells show remarkable multilineage differentiation capacity and direct correlation with intervertebral disc (IVD) degeneration and are therefore an interesting target for regenerative strategies. Nevertheless, there remains controversy over the presence and function of these Tie2^+^ nucleus pulposus cells (NPCs), in part due to the difficulty of identification and isolation.

**Purpose:**

Here, we present a comprehensive protocol for sorting of Tie2^+^ NPCs from human, canine, bovine, and murine IVD tissue. We describe enhanced conditions for expansion and an optimized fluorescence‐activated cell sorting‐based methodology to sort and analyze Tie2^+^ NPCs.

**Methods:**

We present flow cytometry protocols to isolate the Tie2^+^ cell population for the aforementioned species. Moreover, we describe crucial pitfalls to prevent loss of Tie2^+^ NPCs from the IVD cell population during the isolation process. A cross‐species phylogenetic analysis of Tie2 across species is presented.

**Results:**

Our protocols are efficient towards labeling and isolation of Tie2^+^ NPCs. The total flow cytometry procedure requires approximately 9 hours, cell isolation 4 to 16 hours, cell expansion can take up to multiple weeks, dependent on the application, age, disease state, and species. Phylogenetic analysis of the TEK gene revealed a strong homology among species.

**Conclusions:**

Current identification of Tie2^+^ cells could be confirmed in bovine, canine, mouse, and human specimens. The presented flow cytometry protocol can successfully sort these multipotent cells. The biological function of isolated cells based on Tie2^+^ expression needs to be confirmed by functional assays such as in vitro differentiation. in vitro culture conditions to maintain and their possible proliferation of the Tie2^+^ fraction is the subject of future research.

## 1 INTRODUCTION

### 1.1 Development of protocols to isolate nucleus pulposus progenitor cells

**Figure 1:**
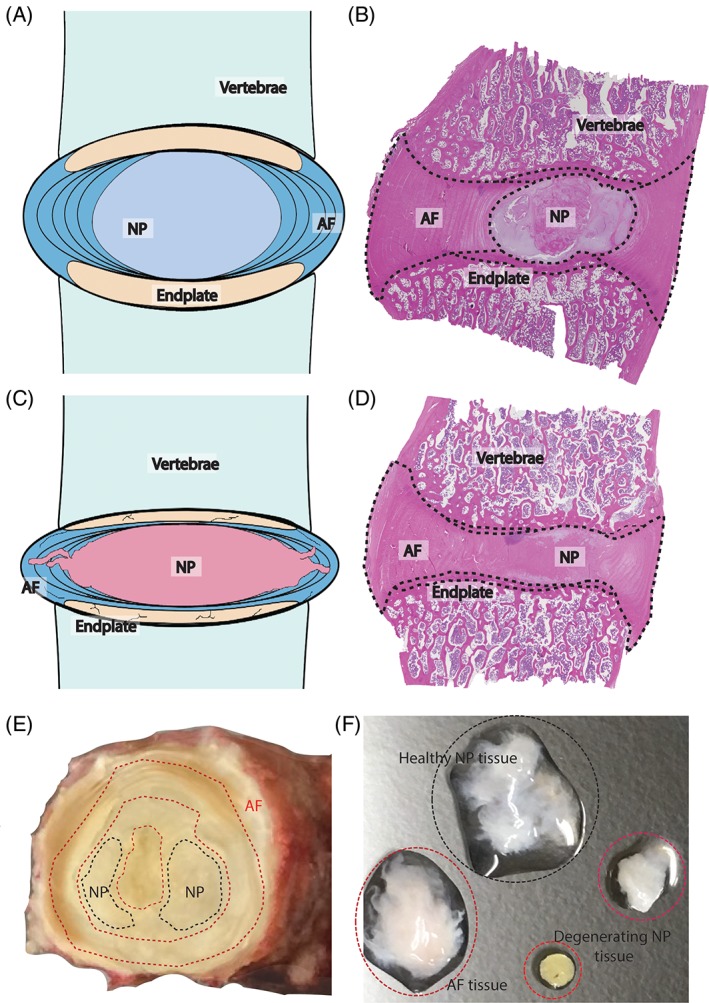
The intervertebral disc and degenerative disc disease. Schematic and a hematoxylin/eosin‐stained histological sections of a healthy (A, B) and degenerated (C, D) canine intervertebral disc. (E) Human lumbar intervertebral disc obtained *postmortem.* Appropriate tissue selection for Tie2 fishing is indicated by dashed black lines, while degenerated or AF tissue areas are indicated by dashed red lines. (F) Human intervertebral disc (IVD)‐derived tissues during discectomy are selected based on morphology, color and stiffness. Only gelatinous, white, and transparent tissue should be used for Tie2 fishing. Yellow and stiff tissue should be disregarded in order to enhance Tie2 detection

Degeneration of the intervertebral disc (IVD) is acknowledged as one main cause of chronic low back and neck pain and poses a significant socioeconomic burden on societies.^1^, ^2^ The healthy IVD is composed of 3 distinct tissue structures, that is, (1) the nucleus pulposus (NP), (2) the annulus fibrosus (AF), and (3) the cartilaginous endplates (EP) bordering the vertebrae (Figure 1A,B). The NP and its cells are derived from the fetal notochord, however with aging the notochordal cell population is replaced by a heterogeneous chondrocyte‐like NPC (NPC) population with distinct functional and phenotypic characteristic. The international spine society has identified a set of markers for the healthy status of the NPCs; for example, FoxF1, Pax‐1, keratin‐8/18 (KRT8/18), carbonic anhydrase‐12, brachyury, galectin‐3, and CD24.^3^ With further aging and degeneration, the active NPCs dedifferentiate and their numbers decrease, altering the extracellular matrix (ECM) composition of the NP; changing from a proteoglycans and collagen type II‐rich gelatinous tissue to a fibrous collagen type I‐rich structure (Figure 1C,D).^4^ The reduced ECM quality results in decreased IVD water retention, tissue flexibility, and mechanical loading capacity along the spine.^5^, ^6^, ^7^

**Figure 2:**
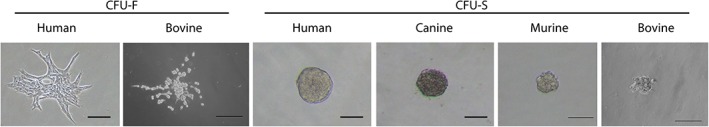
Colony forming units from assessed species. Nucleus pulposus progenitor cells from different species sorted on Tie2 expression were cultured in semi‐solid methylcellulose medium. Fibroblastic colony forming units (CFU‐F) and spherical colony forming units (CFU‐S) emanate as 2 distinguishable colony types. Scale bar represents 50 μm

**Figure 3:**
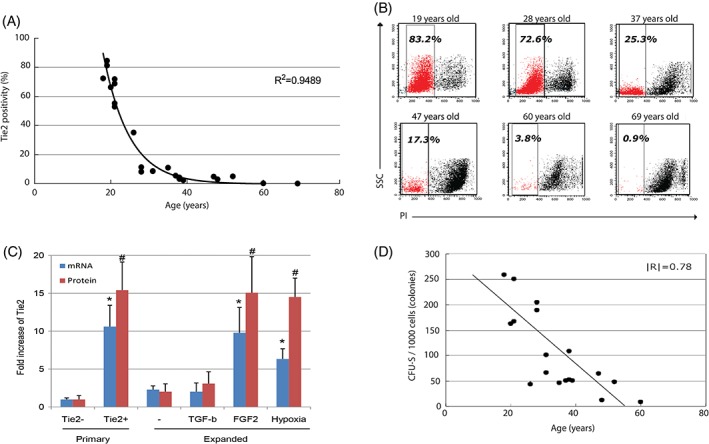
Nucleus pulposus cell viability and Tie2 expression negatively correlates with age. Flow cytometric analysis of human primary nucleus pulposus cells (NPCs) isolated using enzymatic digestion. (A) Percentage of Tie2^+^ NPCs related to age shows a steep decline after 25 years of age (*R* ^2^ = 0.9489). (B) Gating by PI threshold reveals a strong decline in percentage of viable NPCs isolated by enzymatic intervertebral disc (IVD) digestion with age. (C) Tie2^+^ NPCs, isolated by flow‐activated cell sorting for Tie2. Primary refers to NPC that were sorted for negative (Tie2^−^) and positive (Tie2^+^) Tie2 cell populations and were included as controls. Tie2^+^ NPC were expanded in vitro for 7 days (expanded) in αMEM +10% (v/v) FBS in normoxic culture conditions either alone (−), or supplemented with either 10 ng/mL transforming growth factor beta (TGF‐β) or 100 ng/mL fibroblast growth factor 2 (FGF2) or subjected to hypoxic culture conditions. Fold increase in gene (mRNA) and protein (protein) expression levels were determined in Tie2^+^ NPCs by qPCR and flow cytometry, respectively in primary NPCs (Tie2^−^ and Tie2^+^ after sorting) and in expanded Tie2^+^ NPCs. mRNA and protein expression levels in primary Tie2^−^ NPC were set at 1. *P < .05 and ^#^ *P* < .005 as compared to normoxic expanded Tie2^+^ NPCs. (D) the capacity of sorted Tie2^+^ NPCs to form spherical colony forming units (CFU‐S), assessed using methylcellulose semi‐solid culture medium for 10 days, decreases with age

Expression of Tie2/TEK receptor tyrosine kinase (Tie2; also known as CD202) was identified as a marker of local NP progenitor cell population by Sakai et al^8^ in human and murine NPs. These Tie2^+^ NP progenitor cells were characterized by assessment of the total isolated NPC population in methylcellulose semisolid medium, commonly used in colony forming assays (CFA) for endothelial and hematopoietic progenitor cells.^8^, ^9^, ^10^ Two distinct colonies developed, that is, (1) fibroblastic colony forming units (CFU‐F) and (2) spherical colony forming units (CFU‐S), named based on their morphology (Figure 2). High collagen type II and aggrecan protein expression was exclusively observed within CFU‐S. Subsequently, NPCs surface marker was correlated to CFU‐S forming capacity, and strong relationship was observed with Tie2 expression. NPCs were sorted based on Tie2 immunoreactivity and subsequently reassessed by CFA. This resulted in a high frequency of CFU‐S in Tie2^+^ populations but not for Tie2^−^ populations. Notably, Tie2^+^ cells were identified as the precursor that further differentiated and started to express other surface markers, including GD2 (disialoganglioside 2) and CD24.^8^ Moreover, Tie2^+^ cells showed the ability of cell renewal, which is lost with decrease of Tie2 expression.^8^ Next, Tie2^+^ NPCs were assessed on marker expression of macrophage, endothelial cell or pericyte markers (ie, CD11c, CD14, CD31, CD34, CD45, CD144, CD146, and Von Willebrand Factor) to exclude potential contamination, which resulted in no detected immunoreactivity. Also, direct immunostaining against Tie2 in IVD sections revealed distinct Tie2 expression in human and canine NPs (Figure S1, Supporting Information). To further investigate the progenitor cell characteristics of the Tie2^+^ NPCs, differentiation towards osteogenic, chondrogenic, and adipogenic lineages was successfully performed. Moreover, we observed that Tie2 positivity in the NPC population rapidly decreased with progression of IVD degeneration and aging in humans^8^ (Figure 3A) and mice.^11^ Li et al^12^ later demonstrated that despite the relatively low Tie2^+^ NPCs numbers obtainable from degenerated IVD from patients with degenerative IVD disease, proliferation rate, lineage differentiation potency, and regenerative capacity was maintained. Interestingly, these Tie2^+^ NPCs had a superior differentiation capacity towards the chondrogenic lineage compared with bone marrow‐derived mesenchymal stromal cells from the same patients.^12^ Other studies demonstrated that transplanted Tie2^+^ NPCs could be differentiated towards Schwan‐like cells resulting in improved functional recovery in murine sciatic peripheral nerves, again substantiating the progenitor‐like nature.^13^ Nevertheless, Tie2^+^ NPCs application remains limited due to the rapid reduction in ratio of Tie2^+^ NPCs with in vitro expansion (Figure 3B).^8^, ^14^, ^15^ In particular, Tekari et al^15^ showed that Tie2 expression in bovine NPCs decreased from approximately 8% to less than 1% after 2.3 population doublings. Tie2^+^ preservation could, however, be augmented under hypoxic conditions, by supplementation of fibroblast growth factor 2 (FGF2) or synergistic FGF2 and hypoxic conditions (Figure 3C). Rodrigues‐Pinto et al^16^ demonstrated a lack of Tie2 expression during the development of the fetal notochord up to 18 weeks post‐conception, indicating that Tie2 might not play a role during development. Nonetheless, after fetal IVD explantation, isolation, and culture we were able to detect Tie2^+^ NPCs from both human and canine fetal tissue (Figure S1). Cumulatively, these data suggest a role for Tie2^+^ NPCs as an early‐stage response to disruption in the environment to restore homeostasis of the IVD.^8^

Cell‐based strategies for the treatment of chronic back and neck pain due to IVD degeneration have gained significant momentum,^17^ but an optimal cell source remains elusive. Considering their clonal ability for multilineage differentiation potential comparable or even superior to mesenchymal stromal cells,^12^ the Tie2^+^ NPCs can be considered a promising cell transplantation candidate. Tie2^+^ NPCs isolation not only provides an opportunity to study IVD homeostasis and the pathogenesis of IVD degeneration, but it also enables studying the (regenerative) effects of Tie2^+^ NPCs upon other cell residing in the NP, for example, NPCs from degenerated IVDs. Tie2^+^ NPCs or their (recombinantly made) bioactive secreted factors could eventually be applied intradiscally to support IVD regeneration. Thus, there is a need for a methodology to analyze and isolate Tie2^+^ NPCs properly and to enable their application for research and therapeutic purposes. To allow for more reproducible Tie2^+^ NPCs isolation, we here describe extensively an optimized methodology formulated by a multidisciplinary group of experts with experience in Tie2^+^ NPCs research for the harvest, identification, isolation, characterization, and subsequent multiplication of Tie2^+^ NPCs from relevant species commonly applied in IVD research, that is, in human, canine, bovine, and murine origin. We first provide a tissue selection and harvest method protocol, including the pitfalls that could limit Tie2^+^ NPCs yield. Lastly, we provide a complete overview of appropriate gating for FCM and FACS analysis to enhance isolation of the Tie2^+^ NPC population and permit CFU‐S formation.

### 1.2 Experimental design

The Tie2^+^ NPCs isolation protocol is divided into the following sections: (steps 1‐20) selection of appropriate tissue and isolation of total NPC population, (steps 21‐27) optional expansion of NPC, (steps 28‐37) staining for Tie2 and preparation for FCM, (steps 38‐45), data collection by FCM (steps 46‐53), FCM data analysis (steps 54‐69), sorting of Tie2^+^ cells by FACS, and (steps 70‐80) CFA of sorted Tie2^+^ NPCs. All procedures have been successfully tested on IVD samples obtained from fetal‐adult human donors, stillborn‐adult canine donors, and adult bovine and murine donors.

### 1.3 Tissue selection and NPC isolation

The age of the human donor is a crucial indicator of the percentage of Tie2^+^ cells (Figure 3A). Moreover, age also diminishes the number of viable NPCs (Figure 3B), further complicating Tie2^+^ NPCs isolation and analysis. Finally, IVDs afflicted by certain pathologies, such as IVD degeneration, have been shown to correlate with a reduced percentage of Tie2^+^ NPCs.^8^ Thus, for optimal results, young, minimally degenerated IVD samples are applied. For murine^11^ and canine donors, age is also an important negative determinant.

For all species, the tissue is harvested within 1 to 2 days *postmortem* and cell isolation is performed within 24 hours to preserve Tie2^+^ cells and overall cell viability. IVD tissue removed during surgery is kept in serum free medium and stored in a container placed on ice at the day of explantation to preserve cell viability. Alternatively, the IVD tissue can be stored in wet gauzes within a closed container to ensure humidified conditions at 4°C. Prior to cell isolation, it is crucial to establish aseptic working conditions. Within this protocol, solutions are supplemented with penicillin and streptomycin, however additional antibiotics can be considered for tissues derived from less sterile sources, in particular for cadaveric‐derived tissues and primary cultures.

Isolation of NP tissue from the IVD requires careful separation from the surrounding tissue types. In case of full IVD explantation, a cut through the AF is made at the height of the EP, and dismembering one vertebra from the IVD. Thereafter, NP tissue is excised by macroscopic examination. For smaller samples (eg, murine or fetal‐stillborn human and canine tissue), an inverted microscope or binocular glasses are highly recommended. In case of partial IVD explantation, for example, after discectomy, microscopic examination is required to distinguish and separate the gelatinous NP tissue from the fibrous AF. Highly degenerated, fibrotic, or calcified NP tissue segments are avoided. After extraction, large NP samples are minced in approximately 0.3 cm^3^ pieces, while the cells are obtained by enzymatic digestion of the surrounding ECM. It is furthermore recommended to apply specific enzymes and concentrations per species, as the NP tissue size and composition differs considerably between species.^18^ The isolated NPCs can either be analyzed directly (FCM: step 28‐53, FACS: step 54‐69), or expanded first (step 21‐27).

### 1.4 NPC expansion culture

NPC expansion can increase cell numbers for FCM or FACS analysis, which is particularly useful for small tissue sizes or if FACS/FCM analysis is not available immediately after NP digestion. The expansion is ideally performed in αMEM with 10% (v/v) FBS under hypoxic conditions (5% O_2_), to augment maintenance of the Tie2^+^ NPCs phenotype.^12^ Supplementation of 100 ng/mL FGF2, limited culture time and cell passaging can limit the loss of Tie2 expression (Figure 3C).^15^ For murine specifically, it is highly recommended to expand primary isolated cells to induce Tie2 expression.

### 1.5 Tie2 staining and preparation

**Figure 4:**
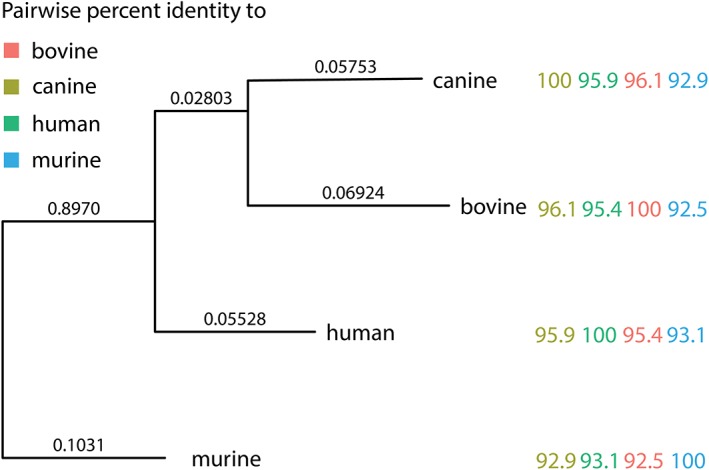
Tie2 protein homology between species. Protein sequences were downloaded from NCBI (murine: NP_038718.2, bovine: NP_776389.1, canine: XP_005626753.1, human: NP_000450.2) and the trees were reconstructed usingphylogeny.Fr using “advanced mode”; aligning the sequences with MUSCLE (v3.8.31) configured for highest accuracy (MUSCLE default settings). The phylogenetic tree was reconstructed using the maximum likelihood method implemented in the PhyML program (v3.1/3.0 aLRT). Graphical representation and edition of the phylogenetic tree were performed with TreeDyn (v198.3). Further analysis was performed in RStudio (v1.0.143) using R (v3.4.1) RC and ggtree (v1.8.1). The pairwise identity is represented as the number of equal residues between pairs

FACS and FCM procedures are prepared with a minimum of 10^3^ cells per condition. For FCM, 3 conditions (Table 1) that is, (1) isotype control, (2) omission of the primary antibody, and (3) Tie2^+^stained cells are required. Homology between canine, human, bovine, and murine Tie2 protein sequence is >92% (Figure 4), and we have successfully assessed a variety of Tie2 antibodies in all mentioned species (Table 2).

**Table 1:** Example of staining conditions of Tie2^+^ cells from the whole NPC population using the anti‐rat Tie2/CD202 antibody on bovine intervertebral disc (IVD) explants

Tube	1	2	3
**Staining with primary antibody**			
Condition	Negative isotype control		Positive control
Primary antibody	Isotype IgG		Tie2 (10 μg /mL)
**Staining with conjugated secondary antibody**			
Condition	Negative isotype control	Negative control	Positive control
Primary antibody	Isotype (10 μg /mL)	*Not applicable*	Tie2 (10 μg /mL)
Secondary antibody	Alexa 488 (10 μg /mL)	Alexa 488 (10 μg /mL)	Alexa 488 (10 μg /mL)
PI	50 μL	50 μL	50 μL

**Table 2:** Overview of antibodies targeting Tie2 among different species

Antibody	Tie2 Santa Cruz clone no.C20 poly Alexa488	Tie2 (3A5) Santa Cruz clone no. sc‐293414	Tie2 R&D clone no.Ms83715 APC	Tie2 R&D clone no.MS83715 PE	Anti‐Tie2/TEK antibody, Millipore clone Ab33	Alexa Fluor® 647 anti‐human CD202b antibody biolegend	Rabbit anti‐rat Tie2/CD202b (Bioss) polyclonal	R&D anti‐mouse Tie2 biotinylated goat polyclonal antibody
Catalog no.	Sc‐324	Sc‐293 414	FAB3131A	FAB3131P	05–584	334 210	Bs‐1300R	BAF007
Human	◯	ND	◯	◯	◯	◯	ND	ND
Canine	◯	☓	◯	◯	◯	☓	ND	☓
Bovine	ND	ND	ND	ND	ND	☓	◯	ND
Murine	ND	ND	ND	ND	ND	ND	ND	◯

◯, indicates successful FCM analysis; ☓, indicates lack of reactivity; ND, indicates that the antibodies reactivity has not been determined towards this species.

### 1.6 FCM and FACS data acquisition and analysis

For this methodology report, FCM and FACS assessment are described for FACS Vantage (BD Bioscience, Erembodegem, Belgium) and data analysis by CellQuest Pro (BD Bioscience) and FlowJo Software (FlowJow LLC, Ashland, OR, US) (version 10.1 for MacOS X, LLC), however LSRII flow cytometer system (Becton Dickinson), FACSCalibur (BD Bioscience), and FACS Diva III (BD Biosciences) have also successfully been applied. FACS Vantage applies a 633‐nm red laser to excite and is reliable at detecting APC‐labels. Researchers employing different machinery should consider the laser and filter wavelengths limits and use appropriate antibody conjugated labels. For FCM and FACS acquisition of NPC and Tie2^+^ NPC gating, the relevant cell population from forward scatter (FSC), side scatter (SSC), and propidium iodide (PI) parameters are crucial. Aged and diseased tissues are associated with a relatively high number of apoptotic cells (Figure 3B) and low Tie2 expression (Figure 3A), making it difficult to distinguish Tie2^+^ cells from the NPC population. First, to detect Tie2^+^ NPCs, it is crucial to limit the FSC/SSC‐based gate to the corresponding NPCs population, disregarding all debris and non‐NPCs. Second, stringent gating is required on PI intensity to disregard all damaged and apoptotic cells. Lastly, after establishing the primary gating threshold for isotype controls and samples, the PI threshold is readdressed to minimize APC positivity in the isotype control. Despite selective screening, it could remain difficult to detect Tie2^+^ NPCs. For samples with low Tie2^+^ frequency, a histogram overview is applied to detect differences in Tie2 intensity between the isotype and Tie2 stained control.

### 1.7 CFA of sorted Tie2^+^ NPCs

CFA is a crucial step to confirm that the Tie2^+^ cells selected from the NPCs are progenitor cells. Tie2^+^ NPCs were discovered based on their capacity to (1) form CFU‐S in a CFA based on methylcellulose semisolid medium, (2) generate GD2 expressing cells, and (3) give rise to a lineage of differentiating NPCs that lose their renewal capacity with loss of Tie2 expression. It is, however, important to recognize that the capacity of Tie2^+^ NPCs to give rise to CFU‐S diminishes with human, canine, and murine donor age (Figure 3D). Tie2^+^ NPCs isolated from adolescent human tissues show a CFU‐S frequency of about 25%, while Tie2^+^ NPCs from older human donors show limited CFU‐S formation. No clear effect on the frequency of CFU‐S formation is observed with days of culture. Finally, in vitro multilineage differentiation potential can be used to confirm the progenitor‐like characteristics of the isolated Tie2^+^ NPCs, following a previously published protocol from Tekari et al.^15^

### 1.8 Level of expertise needed to implement the protocol

For a large volume of juvenile, healthy NP tissue with abundant cell numbers, the described procedures require a basal level of expertise in FCM/FACS methodology, software, and equipment to select, gate appropriately, and analyze the data. For relatively small samples, more degenerated, aged, or otherwise compromised tissue, the selection of proper NP tissue, thereby circumventing concomitant analysis of AF or EP tissue, requires a high level of expertise. Additionally, proper flow cytometric analysis for samples with low cell numbers also requires substantial expertise.

## 2 MATERIALS AND METHODS

### 2.1 Reagents

#### 2.1.1 Cell culture and FCM reagents

- Fresh or cryopreserved human, canine, bovine, or murine NPCs, NP tissue, or IVD tissue.

*Caution*: All experiments should be carried out in a class II biological safety cabinet with proper sterility and antiseptic protocols regarding the preparation of the tissues when derived from cadavers or as surgical waste material. Also, consider protective clothing such a lab coat and gloves. Disposal of biohazardous and human waste material should follow national and institutional protocols.

- ɑMEM (ThermoFisher, Cat. No. 12561056, https://www.thermofisher.com/order/catalog/product/12561056)
- Bovine serum albumin (BSA; Sigma–Aldrich, Cat. No. A9418‐50G, http://www.sigmaaldrich.com/catalog/product/sigma/a9418?lang=ja&region=JP). Store at 4°C.
- Cell banker 1 (Zenoaq, CB011, http://www.zenoaq.jp/cellbanker/ja/cellbanker1.html). Store at 2 to 8°C.
- Chlorhexidine digluconate (Hibiscrub, Cat. No. MRB319, http://www.molnlycke.nl/antiseptica/whole‐body‐wash/hibiscrub/#confirm)
- Collagenase II (260 U/mg; Worthington, Cat. No. LS004176, http://www.worthington-biochem.com/cls/pl.html). Store at 2°C to 8°C, protected from moisture until reconstitution.

*Caution*: Highly toxic; prevent contact or inhalation by appropriate protection. The activity of the collagenase may change from batch‐to‐batch, as such check and adjust the mg employed as necessary to maintain the same levels of activity.

- Collagenase P (Roche, Cat. No. 11‐213‐857‐001, https://roche-biochem.jp/catalog/category_33076/product_3.5.3.3.4.5). Store at −15°C to −25°C in a balanced salt solution.

*Caution*: Highly toxic; Prevent contact or inhalation by appropriate protection.

- EDTA•2Na (DOJINDO, Cat. No. 345‐01865, http://www.siyaku.com/uh/Shs.do?dspCode=W01T02N001)
- Ethanol (VWR, Cat. No. 20816.367, https://nl.vwr.com/store/product/733114/vwrc20816).

*Caution*: Toxic and inflammable; Prevent contact or inhalation by appropriate protection.

- FACS buffer (PBS, 0.5% (v/v) BSA, 1 mM EDTA)
- FBS (ThermoFisher, Cat. No. 16000‐044, https://www.thermofisher.com/order/catalog/product/16000044)
- MethoCult H4230 methylcellulose‐based medium without cytokines for human cells (STEMCELL Technologies, Cat. No. 04230, https://www.stemcell.com/methocult-h4230.html)
- Penicillin/streptomycin (ThermoFisher, Cat. No. 15140122, https://www.thermofisher.com/order/catalog/product/15140122)
- Phosphate buffer saline (PBS‐NaCl; KCl; KH_2_PO_4_; Na_2_HPO_4_ and distilled water) (ThermoFisher, Cat. No. 20012019, https://www.thermofisher.com/order/catalog/product/20012019)
- Pronase (Roche, Cat. No. 11459634001, http://www.sigmaaldrich.com/content/dam/sigma‐aldrich/docs/Roche/Bulletin/1/pronrobul.pdf)

*Caution*: Acute toxicity (oral, dermal, inhalation), Skin and Eye irritation, category 2; Skin sensitization, category 1; prevent contact or inhalation by appropriate protection.

- Recombinant human basic fibroblast growth factor (FGF2; Peprotech, Cat. No. 100‐18B, https://www.peprotech.com/en-GB/Pages/Product/Recombinant_Human_FGF-basic_(154_a.a.)/100-18B)
- Trypan blue (Biorad, Cat. No. 1450021, http://www.bio-rad.com/en-jp/sku/1450021-trypan-blue)

*Caution*: Carc. 1A H350 May cause cancer; wear protective gloves/protective clothing/eye protection/face protection. Dispose of contents/container in accordance with local/regional/national/international regulations.

- TrypLE express without phenol red (Gibco, Cat. No. 12604013, http://www.thermofisher.com/order/catalog/product/12604013)

*Caution*: May cause skin and eye irritation and may be harmful by inhalation or digestion. Prevent contact or inhalation by appropriate protection.

#### 2.1.2 Antibodies and FCM reagents

- Alexa Fluor 647 anti‐human CD202b Antibody (Biolegend, Cat. No. 334210, https://www.biolegend.com/en-gb/products/alexa-fluor-647-anti-human-cd202b-tie2-tek-antibody-5251)
- Allophycocyanin‐conjugated anti‐human Tie2clone No. Ms 83715 (R&D Systems, Cat. No. FAB3131A, https://www.rndsystems.com/products/human‐tie‐2‐apc‐conjugated‐antibody‐83715_fab3131a)
- Allophycocyanin‐conjugated streptoavidin (BD Biosciences, Cat. No. 349024, http://www.bdbiosciences.com/us/reagents/research/antibodies‐buffers/second‐step‐reagents/avidinstreptavidin/apc‐streptavidin/p/349024)
- Anti‐mouse Tie2 biotinylated goat polyclonal antibody (R&D Systems, Cat. No. BAF007, https://www.rndsystems.com/products/goat-anti-mouse-igg-biotinylated-antibody_baf007)
- Anti‐Tie2/TEK Antibody, Millipore clone Ab33 (MERCK, Cat. No. 05‐584, http://www.merckmillipore.com/NL/en/product/Anti‐Tie2/TEK‐Antibody‐clone‐Ab33,MM_NF‐05‐584)
- BD FACS clean solution (Becton Dickinson, Cat. No. 340345, http://www.bdbiosciences.com/jp/services/ordersupport/information/20141107.jsp)

*Caution*: Causes skin irritation, severe eye irritation, it toxic to aquatic life. Wash thoroughly after handling. Avoid release to the environment. Wear protective gloves/eye protection/face protection.

- BD FACS clean solution (Becton Dickinson, Cat. No. 340346, http://www.bdbiosciences.com/jp/services/ordersupport/information/20141107.jsp)
- BD FACSFlow sheath fluid (Becton Dickinson, Cat. No. 342003, http://www.bdbiosciences.com/jp/services/ordersupport/information/20141107.jsp)

*Caution*: Highly toxic; prevent contact or inhalation by appropriate protection.

- Goat anti‐mouse IgG FITC; Goat Anti‐Mouse (BD Biosciences, Cat. No. 349031, http://www.bdbiosciences.com/us/applications/research/b‐cell‐research/immunoglobulins/mouse/fitc‐goat‐anti‐mouse‐ig‐polyclonal/p/349031)
- Goat anti‐rabbit IgG (H + L) Alexa 488 conjugate (Invitrogen, Cat. No. A‐11008, 2 mg/mL, https://www.thermofisher.com/antibody/product/Goat-anti-Rabbit-IgG-H-L-Cross-Adsorbed-Secondary-Antibody-Polyclonal/A-11008)
- Human/mouse Tie‐2 biotinylated antibody (R&D systems, Cat. No. BAF762, https://www.rndsystems.com/products/human-mouse-tie-2-biotinylated-antibody_baf762)
- Propidium iodide staining solution (BD Pharmingen Cat. No. 556463, 30 μg/mL, http://www.bdbiosciences.com/us/applications/research/apoptosis/buffers‐and‐ancillary‐products/propidium‐iodide‐staining‐solution/p/556463)
- Rabbit anti‐rat Tie2/CD202b (Bioss antibodies, Cat. No. bs‐1300R, 1 mg/mL https://www.biossusa.com/products/bs‐1300r.html)
- Rabbit IgG isotype control (Invitrogen, Cat. No. 10500C, 3 mg/mL, https://www.thermofisher.com/antibody/product/Rabbit-IgG-Isotype-Control/10500C)
- Tie2 (3A5) Santa Cruz clone No. sc‐293414 (Santa Cruz, Cat. No. Sc‐293414, https://www.scbt.com/scbt/product/tie‐2‐antibody‐3a5)
- Tie2 Santa Cruz clone No. C20 poly Alexa488 (Santa Cruz, Cat. No. Sc‐324, https://www.scbt.com/scbt/product/tie-2-antibody-c-20?productCanUrl=tie-2-antibody-c-20&_requestid=3764108)

### 2.2 Equipment

- 0.22‐μm membrane filter (Techno Plastic Products AG, Cat. No. 99722, http://www.tpp.ch/page/produkte/12_filtration_spritzenfilter.php)
- 0.45‐μm membrane filter (Techno Plastic Products AG, Cat. No. 99745, http://www.tpp.ch/page/produkte/12_filtration_spritzenfilter.php)
- 10 cm^2^ ø petridishes (Greiner Cellstar, Cat. No. 664160, https://shop.gbo.com/en/row/products/bioscience/cell‐culture‐products/cellstar‐cell‐culture‐dishes/standard‐cell‐culture‐dishes/664160.html)
- 1‐mL syringe needleless (Terumo. Cat. No. SS‐01 T, https://www.terumo.co.jp/medical/equipment/me05.html)
- 15‐mL conical tubes (Greiner Cellstar, Cat. No. 188271, https://shop.gbo.com/en/row/products/bioscience/tubes‐beakers/tubes/15ml‐cellstar‐polypropylene‐tube/188271.html)
- 30‐mL syringe needleless (Terumo. Cat. No. SS‐30ESZ, https://www.terumo.co.jp/medical/equipment/me05.html)
- 35‐mm^2^ petridishes (STEMCELL Technologies, Cat. No. 27100, https://www.stemcell.com/35-mm-culture-dishes.html)
- 50‐mL conical tubes (Greiner Cellstar, Cat. No. 227261, https://shop.gbo.com/en/row/products/bioscience/tubes-beakers/tubes/50ml-cellstar-polypropylene-tube/227261.html)
- 5‐mL polystyrene round bottom tubes (BD Falcon Cat. No. 352058, https://catalog2.corning.com/LifeSciences/en-US/Shopping/ProductDetails.aspx?productid=352058(Lifesciences])
- 5‐mL polystyrene round bottom tubes with cell strainer snap cap 35 μm polystyrene mesh (Corning, Cat. No. 352235, https://catalog2.corning.com/LifeSciences/en‐US/Shopping/ProductDetails.aspx?productid=352235(Lifesciences])
- 6‐wells plates (Greiner Cellstar, Cat. No. 657160, https://shop.gbo.com/en/row/products/distribution/5_0060/5_0060_0020/5_0060_0020_0020/657160.html)
- CellQuest Pro. (BD Bioscience)
- Cell strainers 40 μm (Falcon, Cat. No. 087711, https://www.fishersci.com/shop/products/falcon-cell-strainers-4/p-48680)
- Curettes size A (World Precision Instruments, Cat. No. 501773, https://www.wpiinc.com/products/laboratory‐supplies/501773‐meyhoefer‐curette‐14cm‐1.5mm‐diameter)
- Electronic analytical balance (eg, Satorius, Cat. No. ED124S, https://www.coleparmer.com/i/sartorius‐ed124s‐extend‐ed‐analytical‐balance‐120g‐x‐0‐1‐mg/1121903)
- Inverted microscope (eg, Olympus, Cat. No. IX70)
- Laboratory centrifuge (eg, SIGMA, Cat. No 4K15)
- Microflex XCEED powder‐free surgical gloves (Microflex, https://www.fishersci.com/shop/products/microflex-xceed-powder-free-nitrile-examination-gloves-5/p-4099926)
- Multigas thermal incubator, set to 37°C, 5% CO_2_, 2% O_2_ (eg, ASTEC, Cat. No. CDI‐325, http://www.astec-bio.com/global/cell/a02/index.html)
- Neubauer cell counting chamber set (eg, ERMA Tokyo, 03‐200‐1 to 03‐200‐4, http://www.erma.co.jp/product/bloodtest/bloodboard/88/)
- Serological pipette 10 mL (SARSTEDT, Cat. No. 86.1254.001, https://www.sarstedt.com/en/products/laboratory/liquid-handling/serological-pipettes/product/86.1254.001/)
- Serological pipette 25 mL (SARSTEDT, Cat. No. 86.1685.001, https://www.sarstedt.com/en/products/laboratory/liquid‐handling/serological‐pipettes/product/86.1685.001/)
- Sterile disposable surgical blades 20 (Swann‐Morton, Cat. No. 0206, http://www.swann-morton.com/product/36.php)
- Sterile forceps (World Precision Instruments, Cat. No. 14226, https://www.wpiinc.com/products/laboratory‐supplies/14226‐adson‐forceps‐12cm‐straight‐serrated)
- Sterile scissors (World Precision Instruments, Cat. No. 501225, https://www.wpiinc.com/products/laboratory‐supplies/501225‐operating‐scissors‐16cm‐sharp‐sharp‐straight)
- Sterile surgical scalpel ELP handle number 10 (Axel, 2‐5726‐01, https://axel.as-1.co.jp/asone/d/2-5726-01/?cate=D)
- Sterile surgical scalpel ELP handle number 20 (Axel, 2‐5726‐06, https://axel.as-1.co.jp/asone/d/2-5726-06/?cate=D)
- Sterile tweezers (World Precision Instruments, Cat. No. 501975, https://www.wpiinc.com/products/laboratory‐supplies/501975‐economy‐tweezers‐2‐11cm‐0.4x0.55mm‐tips/)
- Surgical gloves (Gammex, http://www.ansell.com/en/Brands/Gammex/Surgical%20Gloves/gammex-latex-chemo)
- T25 flasks (Greiner Cellstar, Cat. No. 690175, https://shop.gbo.com/en/row/products/bioscience/cell‐culture‐products/cellstar‐cell‐culture‐flasks/filter‐cap‐cell‐culture‐flasks/690175.html)
- T75 flasks (Greiner Cellstar, Cat. No. 658175, https://shop.gbo.com/en/row/products/bioscience/cell‐culture‐products/cellstar‐cell‐culture‐flasks/filter‐cap‐cell‐culture‐flasks/658175.html)
- Thermal incubator with shaker (eg, AS ONE, Cat. No. 1‐6142‐01, https://axel.as-1.co.jp/asone/d/1-6142-01/)

### 2.3 Procedure

*Caution*: Before collection and use of human tissue samples, consider national legislation regulating the need for patient informed consent on collection and usage of donor tissue material and the requirement of approval of ethical committees for collection and use of human‐derived tissue materials.

*Caution*: The use of (experimental) animals should be reviewed and approved by ethical committees responsible for oversight in care and use of experimental animals in your institutions according to (inter)national legislation before initiation of the procedures. In certain circumstances, when the material is obtained as cadaver or where animal tissue is obtained from third parties (eg, samples from abattoir, tissues derived from other unrelated experiments and where no handling of any live animals has occurred to obtain the tissue samples or cadavers) a full ethical consideration may not be required. However, it is recommended to contact the local ethics office/animal welfare body for determination as to whether or not the application for exemption is appropriate.

#### 2.3.1 Nucleus pulposus cell isolation—Timing 5 to 10 hours

*Critical*: The protocol as described below is specified for moderately degenerated, adult human IVD tissue. Modifications of the protocol to address species or age‐ and disease‐related alteration are specified or mentioned in “critical” sections.

*Caution*: Since tissue samples can be carriers of infectious diseases, ensure sufficient protection (ie, lab coat and surgical gloves) to prevent potential contact with tissue sample, blood, and other sources of contamination.

1. Retrieve complete IVD explant and store in wet gauzes in a closed container to ensure humidified conditions until use and keep on ice. Surgically removed sectioned NP tissue should be stored in a sealed container submerged in sterile saline solution, such as PBS. Place on ice until use. Register (anonymously) accompanying information, for example, species, sex, age, time of death, cause of death, IVD location within the spine, spine‐related diseases, grade of degeneration, and reason for IVD explantation.

*Critical*: Do not use tissues later than 48 hours *postmortem*, as this severely diminishes cell viability and Tie2 expression.

2. Under aseptic conditions, place the retrieved tissue in a 10‐cm ø petridish and carefully wash it with 10 mL of PBS solution. Repeat the washing step until all blood is removed from the tissue. In case of intact IVD explants, the exterior is ideally washed with chlorhexidine digluconate (2 × 1 minute). Alternatively, the tissue can be incubated for 5 minutes in 70% (v/v) ethanol.
3. Remove muscle, fat, and nerve tissue surrounding the IVD with sterile surgical forceps, tweezers, scissors, and scalpels. In case of an intact IVD tissue explant, make sure not to puncture the AF during this procedure. Detach the IVD from the vertebrae using a sterile scalpel.

*Critical*: Refresh the scalpel blade after detachment of the IVD from the vertebrae, to prevent introducing infectious agents from the exterior to the NP. Ideally, and dependent on the size of the samples, the dissection starts with a blade no. 20 to remove large pieces of tissues. After that, continue with the meticulous dissection employing disposable blades no. 10.

4. Isolate the gelatinous NP from surrounding AF tissue with macroscopic or microscopic examination using clean, sterile equipment.

*Critical*: In case of burst fracture samples where the upper EP is damaged or connected to a fractured vertebral body, NP tissue should only be harvested on the side of the intact opposing EP.

*Critical*: In case of Pfirrmann grade III to IV discs for which it is difficult to distinguish between the NP and inner AF, only central NP tissue should be harvested to avoid possible contamination of other tissue types. For excised IVD tissue from surgery, NP tissue that cannot be clearly distinguished as NP tissue should be excluded. Also, detach denatured NP sites from the gelatinous NP. Discriminate nondenatured NP tissue based on a transparent or white translucent appearance with low stiffness (Figure 1E,F).

*Critical*: In case of very small IVD volumes, that is, murine and fetal‐stillborn human or canine tissue, it is recommended to use an inverted microscope or binocular glasses to be able to separate NP tissue from AF, EPs, and vertebrae. Note that in very young individuals, the vertebrae are not entirely calcified, making it challenging to distinguish them from other tissue types. The use of a small curette (size A) is recommended to separate the brittle, loose NP tissue from the firmer AF tissue and EPs.

(a) For fetal and stillborn samples, wash the collected NP tissue in a 15‐mL tube with αMEM and centrifuge at 500*g* for 5 minutes at room temperature. Thereafter, go immediately to step 12a, since enzymatic digestion with Pronase considerably decreases NPC viability and is not necessary because of the loose ECM. Tissue digestion should be reduced to a minimum in these samples.

• *Pause point*: Place separated NP tissue in excessive serum‐free medium (dependent on NP volume, minimally 10 mL) in a 15‐ or 50‐mL conical tube on ice and store at 4°C for maximally 24 hours.

5. In case of large NP volumes, mince the isolated NP tissues to approximately 0.3 cm^3^ fragments on a 35‐mm or 10 cm ø petridish with a fresh, sterile scalpel blade no. 10.
6. Transfer the (sectioned) NP fragments in a 10‐mL PBS‐containing 50 mL conical tube (weighted prior to NP tissue collection) and determine the total NP tissue wet weight. In case of smaller NP tissue yield (≤2 g), use a 15‐mL conical tube. Store the tissue suspension on ice.
7. Dissolve the collagenase P in αMEM with 10% (v/v) fetal bovine serum (FBS) solution to a concentration of 0.025% (w/v). Filter the solution with a 0.45‐μm membrane filter and add the filtered solution. Store the solution on ice.

(a) For bovine NP tissue, prepare 1.9% (w/v) of Pronase (dissolved in PBS) and filter the solution with a 0.22‐μm membrane filter. Store on ice until use.

*Caution*: Collagenase P and Pronase are harmful; prevent contact or inhalation by appropriate protection.

*Critical*: Make sure that calcium ions are present in the buffer solution. FBS is employed to mitigate enzymatic activity to limit cell damage during digestion. Depending on the FBS batch the w/v FBS present may need to be adjusted as it may diminish the activity of the digesting enzymes.

8. Centrifuge the NP tissue suspension from step 6 at 500*g* for 5 minutes at room temperature and gently discard the supernatant. Add 10 mL 1 × TrypLE Express to the NP tissue suspension.
9. For bovine NP tissue, incubate the isolated NP tissue in the Pronase solution for 1 hour at 37°C and 5% CO_2_. Gently shake the tissue suspension with a shaker at approximately 60 shakes/min in a 37°C enclosed environment for 30 to 60 minutes. Terminate the incubation when tissue pieces start to disintegrate.

*Critical*: Do not incubate TrypLE Express longer than 1 hour. In case of small tissue yield (<1 g) incubate for 15 minutes and microscopically examine the state of digestion. For relatively large NP tissue sections, we recommend dividing the tissue over multiple tubes.

10. Centrifuge the tissue at 500*g* for 5 minutes at room temperature.
11. Wash the tissue twice by adding 25 mL of PBS and centrifuge the sample at 500*g* for 5 minutes at room temperature.
12. Discard the supernatant and add 25 mL (for NP tissue >2 mg) or 10 mL (for NP tissue <2 mg) collagenase P solution prepared in step 7 by decantation. (See Table 3 for troubleshooting solutions.)

**Table 3:** Troubleshooting for the procedure

Step	Problem	Possible reason	Solution
12	Poor digestion of NP tissue	Inactive enzymes	Store enzymes properly and in appropriate aliquots
			Prevent freeze–thaw cycles of enzymes
		Inadequate Ca^2+^concentrationin collagenase suspension	Supplement the collagenase solution with additional 5 mM Ca^2+^
		Insufficient enzymes	Increase the concentration of enzymes
		Tissue pieces are too large	Cut the tissue into smaller sections
19	Aggregation of cells after digestion	Secreted DNA by necrotic cells, which stimulate cell aggregation	Wash tissue prior to digestion with PBS
			Reduce agitation by adding DNAse
			Reduce enzyme concentration and enzyme incubation time
	Fraction of viable cells is too small	Excess protease/enzyme digestion	Reduce enzyme concentration and enzyme incubation time
			Add albumin or heated serum to cell suspensions to decrease agitation
		pH change	Add buffer (e.g. HEPES) to solution
			Aerate solution during digestion
			Lower digestion incubation time by increasing enzyme concentration
			Increase the ratio of enzyme containing medium to during tissue digestion
		Oxygen tension drops	Lower digestion incubation time by increasing enzyme concentration
			Increase the ratio of enzyme containing medium to during tissue digestion
			Aerate solution during digestion
20	No/very low number of viable cells present after enzymatic digestion	Cell damage by enzymatic digestion (in particular for fetal cells)	Lower the enzyme concentration and/or enzymatic digestion time.
			In very gelatinous NP tissue, the pronase step could be reduced/omitted.
	No/very low number of viable cells present after cryopreservation	Cell damage by cryopreservation (in particular for fetal cells)	Culture cells for about 7 days and analyze or cryopreserve thereafter.
44	NPC population cannot be recognized	Cell suspension contains high numbers of dead cells, tissue debris, and non‐NPC.	To remove erythrocytes and tissue and cell debris, lymphoprep (StemCell Technology, Cat. No. 07851) can be applied.
		Limited NPCs are present	Set the FSC‐H and SSC‐H gate over a larger region that captures 500 000 cells and narrow the gate later during the procedures to further specify the correct NPC population.
		Release of antibody by harsh and repeated washing (in particular observed for fetal samples)	Limit the mechanical force generated during the washing step with FACS buffer, which might separate the antibody from its antigen, by gently adding the solutions via the inner wall of the tube in a slightly tilted position.
			Reduce the number of washing repetitions during the staining procedures
52	Tie2 positive cells cannot be detected	The examined tissue is derived from aged, diseased, or degenerated tissue.	Do not digest tissue, but allow cells to be cultured inside their tissue sections prior to enzymatic digestion.
65	Cell sorting results in continuous clogging of the machinery flow path	Aggregation of the NPC	Limit the time of storing the cell suspension on ice prior to sorting
			Dilute the concentration of NPC suspension to limit the rate of aggregation
			Re‐filter the cells using the cell strainer cap used in step 56 and 57
67	NPC population cannot be recognized	Cell suspension contains high numbers of dead cells, tissue debris, and non‐NPC.	To remove erythrocytes and tissue and cell debris, lymphoprep (StemCell Technology, Cat. No. 07851) can be applied.
		Limited NPCs are present	Set the FSC‐H and SSC‐H gate over a larger region that captures 500 000 cells and narrow the gate later during the procedures to further specify the correct NPC population.
		Release of antibody by harsh and repeated washing	Limit the mechanical force generated during the washing step with FACS buffer, which might separate the antibody from its antigen, by gently adding the solutions via the inner wall of the tube in a slightly tilted position.
			Reduce the number of washing repetitions during the staining procedures

(a) For fetal and stillborn samples, add 5 mL 0.03% (w/v) collagenase II dissolved in αMEM (filtered with a 0.22‐μm membrane filter).

(b) For bovine NP tissue, add 25 mL 0.012% (w/v) collagenase II dissolved in αMEM (filtered with a 0.22‐μm membrane filter).

*Caution*: Collagenase P and II are harmful; prevent contact or inhalation by appropriate protection.

*Critical*: For fetal and stillborn samples, enzymatic Pronase pre‐treatment or a higher collagenase II concentration considerably decreases cell viability and is therefore avoided.

13. Gently shake the tissue suspension with a shaker at 37°C for 60 to 120 minutes. Terminate the enzymatic digestion when the tissue pieces have entirely disintegrated.

(a) For fetal and stillborn samples, incubate 15 to 30 minute. After that, mix by inversion to disintegrate small tissue clumps.

(b) For bovine NP tissue, incubate NP overnight (12‐16 hours) instead of 60 to 120 minutes.

14. Decant the cell suspension onto a 40‐μm cell strainer on a 50‐mL tube, removing undigested pieces of tissue by filtration. Discard the strainer.

(a) For fetal and stillborn samples, skip this step to prevent further damage to the low cell numbers.

15. Wash the NPCs with αMEM supplemented with 10% (v/v) FBS to inactivate the enzymes.
16. Centrifuge the cell suspension at 500*g* for 5 minutes at room temperature. Carefully discard the supernatant by decantation.

(a) For fetal and stillborn samples, proceed with step 20: immediately expand the cells because of low cell numbers (<10^3^ viable cells).

17. Wash the cell suspension twice in 10 mL PBS and centrifugation at 500*g* for 5 minutes at room temperature.
18. Add 500 to 2000 μL αMEM and resuspend the cells by gently tapping the tube (if the cell volume is large, use a 200‐μL pipette to resuspend the cells).
19. Count and calculate the number of viable and dead cells in the suspension, for example, by using trypan blue mediated counting method. (See Table 3 for troubleshooting solutions.)

(a) Fetal and stillborn samples are shortly digested to remove the loose ECM. Large vacuolated notochordal cells are typically present in these samples, and the digestion protocol does not dissociate the notochordal cell clusters into single singles. As such, cell counting is not reliable with a trypan blue‐based automated cell count and viability. When sufficient sample is available, consider employing a destructive technique in an aliquot of a sample based on PI and a Nucleocounter (NC‐100; Chemometec, Nieuwegein, The Netherlands). Fluorescent PI can bind double‐stranded DNA but is unable to permeate the membrane of living cells. In this assay, the number of viable cells is determined by calculating the difference between the number of dead cells in suspension before (dead cell concentration) and after lysis of the cell membranes (total cell concentration, including clustered cells).^19^

*Critical*: Cell count and cell viability with trypan blue exclusion in an automated cell counter is reliable only in single cell solution and in the absence of the typically vacuolated notochordal cells.

20. Add 10 mL of 10% (v/v) FBS, 100 U/mL, penicillin 100 μg/mL, streptomycin αMEM to a 10‐cm ø petridish and add 1.0 × 10^5^ viable cells to each plate. After adding the cells, swirl the plate several times to distribute the cells equally. If the number of collected viable cells is less than 10^5^, transfer the cell suspension into a 6‐well plate well filled with 2 mL of αMEM containing 10% (v/v) FBS. (See Table 3 for troubleshooting solutions.)

• *Pause point*: Dispersed NPCs derived from adult IVD tissues can be cryopreserved at −196°C in αMEM +10% (v/v) FBS + 10% (v/v) DMSO until use.

*Critical*: Nonpassaged fetal‐stillborn human and canine NPCs cannot be cryopreserved, since cell viability decreases considerably. Therefore, expand these NPCs one passage before cryopreserving them.

*Critical*: Cryopreservation has been shown to reduce the number of Tie2^+^ NPCs.

#### 2.3.2 Expansion of NPCs (optional)—Timing 7 days

*Critical*: Ideally, fresh, primary NPCs are used for Tie2 analysis and sorting. Only when cell numbers are very low (<10^3^ cells), expand the NPCs in well‐defined culture conditions in hypoxia (5% O_2_) to maintain Tie2^+^ cell numbers. Optionally, the cell culture can be supplemented with FGF2 (100 ng/mL). Limit the number of passages to maximally 2 cell passages. In case of <10^3^ cells, limit passaging to only once, to prevent loss of Tie2 expression.

21. Incubate the NPCs at 5% O_2_, 5% CO_2_, 37°C to about 70% confluency in ɑMEM containing 10% (v/v) FBS, 100 U/mL penicillin, 100 μg/mL streptomycin in a 10‐cm ø petridish or T75 flask (adult human, canine, bovine), 6‐well plate, or T25 flask (stillborn‐fetal samples). NPCs generally reach 70% confluency in about 1 week after seeding.

*Critical*: NPCs derived from aged patients show low proliferative potential. Do not expand cells longer than 1 month.

*Critical*: Vacuolated notochordal cells will adhere to the plate either as single cells or in clusters and will gradually lose their vacuolated phenotype over the period of 7 to 10 days.^20^

22. Upon 70% to 80% confluency, aspirate and discard the culture medium and rinse the cell layer with 10 mL of PBS. Subsequently, discard the PBS and cover the monolayer culture with 5 mL (10 cm ø petridish, T75 flask) or 2 mL (6‐well plate, T25 flask) 1× TrypLE Express.
23. Incubate the plate/flask at 37°C, 5% O_2_, 5% CO_2_ for 5 minutes and confirm detachment by microscopy.
24. After detachment, transfer the cells to a 15‐mL sterile conical tube with αMEM +10% (v/v) FBS and centrifuge the cell suspension at 500*g* for 5 minutes at room temperature.
25. Discard the supernatant by decantation and resuspend the cells in 1 to 3 mL αMEM +10% (v/v) FBS (volume dependent on expected cell numbers; 1 mL for T25 flask and 6‐wells plate and 2 to 3 mL for T75 flask and 10 cm ø petridish) and mix by inversion.
26. Count and calculate the number of viable and dead cells using Trypan blue.
27. For use in antibody staining follow procedures from step 28. For continuous expansion, repeat step 21 to 26.

• *Pause point*: Passaged cells can be cryopreserved at −196°C in αMEM +10% (v/v) FBS + 10% (v/v) DMSO until further analysis.

*Critical*: Cryopreservation has been shown to reduce the number of Tie2^+^ NPCs.

#### 2.3.3 Antibody staining—Timing 1 to 2 hours

28. Label 3 polystyrene round bottom tube according to Table 1. Transfer 5.0 × 10^4^ cells to each tube and add 2 mL of FACS buffer.

*Critical*: Total cell number used for FCM and FACS analysis should minimally comprise a total of 5.0 × 10^3^ cells and maximally 1.0 × 10^5^ cells condition

29. Centrifuge the cell suspension obtained from step 28 at 500*g* for 5 minutes at 4°C and discard the supernatant by decantation.
30. Resuspend the cell pellet in 50 μL FACS buffer.
31. Add 5 μg to the 50 μL cell suspension of anti‐human Tie2 to FACS tube #3 following Table 1. Mix gently with a 200‐μL pipette. Add 5 μL isotype IgG (corresponding to the species from which the anti‐Tie2 antibody is derived from) to the 200 μL cell suspension following table for the isotype control to tubes #1 following Table 1.

(a) For canine samples add 3 μL of 1 mg/mL Bioss antibodies Rabbit anti‐rat Tie2/CD202b to the tube #3.

(b) For bovine samples, add 1 μL of 1 mg/mL Bioss antibodies Rabbit anti‐rat Tie2/CD202b rabbit polyclonal antibody to 100 μL cell suspension. For the control, rabbit IgG isotype is added in the same concentration as the Tie2 antibody.

(c) For mouse samples add 5 μL of Goat Anti‐Mouse IgG Biotinylated Antibody to tube #3.

*Critical*: As the number of Tie2^+^ cells is low and the relative expression is weak, it is recommended to increase the antibody concentration according to the number of cells. Incubate the cell suspension on ice protected from light for 30 minutes.

32. Add 2 mL of FACS buffer and centrifuge the cell suspension at 500*g* for 5 minutes at 4°C. Discard the supernatant by decantation. Repeat twice.
33. Resuspend the pellets in 200 μL of FACS buffer to each tube (Table 1). Continue to step 38 when no secondary antibody is applied.

(a) For canine samples, add 3 μL of Alexa 488 goat anti‐rabbit antibody.

(b) For bovine samples, resuspend the cell pellet in 200 μL FACS buffer and add 1 μL of Alexa 488 goat anti‐rabbit antibody (10 μg/mL).

*Critical*: Assure that when applying a secondary antibody, the antibody is not reactive to the species of the tissue material, and is reactive to species from which the primary antibody was derived.

34. Leave on ice, kept from light, for 30 minutes.
35. Centrifuge samples at 500*g* for 5 minutes at 4°C. Remove the supernatant by decantation and add 2 mL of FACS buffer. Repeat the washing procedure twice.
36. Remove supernatant and add 200 μL of FACS buffer and gently resuspend the entire cell pellet.
37. Add 50 μL (30 μg/mL) of PI solution.

*Caution*: PI is carcinogenic, wear gloves and handle with care.

*Critical*: Start analysis by FCM or FACS as quickly as possible. For storage, samples should be kept on ice kept from light, but not longer than 8 hours.

#### 2.3.4 Data collection by FCM—Timing 2 hours

38. Turn on the flow cytometer and perform normal fluid management setup according to manufacturers' instructions.
39. Start CellQuest Pro (or other FCM affiliated software) and open a new file (or a previously saved formatted file and switch all the acquisition plots to analyze).
40. Create dot‐plots of FSC‐H vs SSC‐H (overall definition of the cell population), dot plots of SSC‐H vs FL‐3‐H (PI) (rough live/dead discrimination), SSC‐H vs FL‐4‐H (APC) (measurement of Tie2), and dot plots of SSC‐H vs FL‐4‐H (APC‐control) (Figure 6).
41. Connect to the cytometer and connect appropriate instrument settings: FSC (Voltage E‐1, Ampgain 7.21, Linear mode) SSC (Voltage 271 to 358, Ampgain 1.00, Linear mode) primary threshold parameter: FSC, value 162, secondary threshold parameter: none. FL‐4 (Voltage 678 to 704, Ampgain 1.00, Logarithmic mode).
42. Attach the APC‐C sample and preview the voltage by running at the slowest flow rate (Sheet pressure 4.5 PSIG, sample pressure 5.0 PSIG, 12 μL/min). If required, adjust the voltage of the FSC, SSC, PI, and APC to ensure all the measured dots are within the plot.

*Critical*: In case of low cell numbers present in the samples, prevent extended use for setting up the correct voltage to prevent loss of cells.

43. Attach the Tie2 stained sample and preview the voltage settings for appropriate APC detecting, by ensuring all the dots are within the borders of the plot.

*Critical*: In case of low cell numbers prevent extended use for setting up the correct voltage

44. Measure all samples using the established settings. (See Table 3 for troubleshooting solutions.)
45. When all samples are analyzed, and data storage is completed, perform fluid management and cleaning as instructed by the manufacturer and progress to data analysis.

#### 2.3.5 Data analysis and Tie2 detection—Timing 2 hours

**Figure 5:**
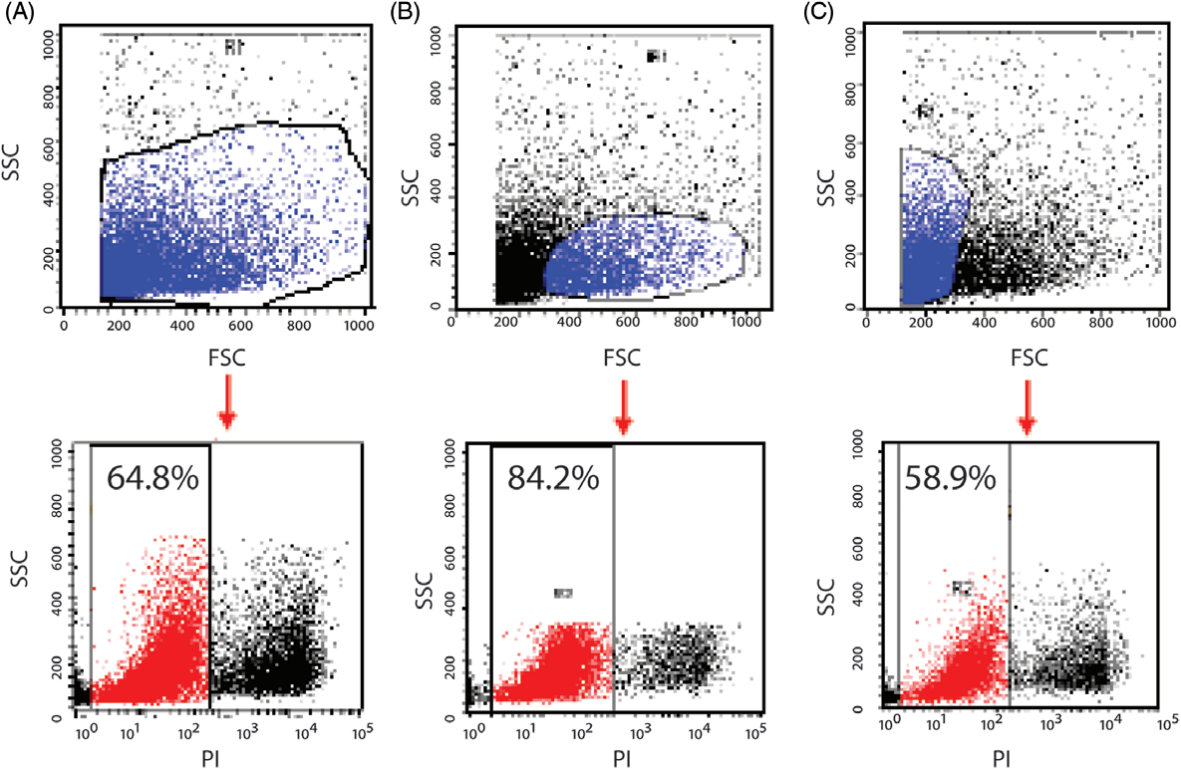
Selective forward scatter (FSC) and side scatter (SSC) gating is crucial for viability examination. After enzymatic digestion and filtration, human nucleus pulposus cell suspensions contain connective tissue fragments, granules of cellular components and/or dead cells. This is different from hematopoietic cells or tissue types that are dissociated easily from their extracellular matrix. The percentage of dead cells determined by propidium iodide staining will differ by gating and thus, FSC and SSC measures are essential to exclude these components. Contamination of noncellular components and dead cells will result in undesirable nonspecific staining

**Figure 6:**
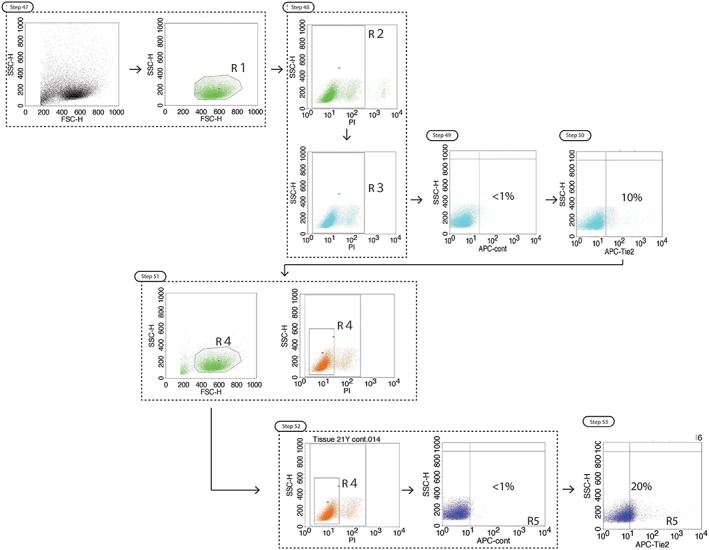
Step wise overview of FCM gating. Schematic overview of procedural steps for appropriate gating of Tie2^+^ NPCs. Percentages indicate the positivity for Tie2 in the particular gate

46. Start CellQuest Pro and open a new file (or a previously saved formatted file and switch all the acquisition plots to analyze).
47. Import a dot plot of FSC‐H against SSC‐H. Create a polygon gate (R1) in linear mode with an SSC‐H of about 150 to 400 and FSC‐H of about 250 to 800 to select the NPC population (Figures 5 and 6).
48. Import a dot plot of SSC‐H vs FL‐4‐H (PI). To gate the viable cells of the selected NPC population gate (R2) by square mode the entire population of cells that is grouped with low PI fluorescence intensity, commonly between 5 and 150 (Figure 6). A gate (R3) containing both the gates of R1 and R2 is automatically created in the gate list.
49. Import a dot plot of SSC‐H vs FL‐5‐H (APC Tie2) and connect this plot to the R3 gated cell population. Import the data of the isotype control. Employ a quadrant state in the dot plot and adjust the position of the threshold so that the percentage in the left bottom quadrants is equal or larger than 99% (indicating negative for Tie2) while the threshold holds the lowest obtainable intensity (Figure 6).

*Critical*: Ensure that gate R5 includes more than 1000 analyzed events in order to draw a conclusion.

50. Import the dot plots of SSC‐H vs isotype control FL‐5‐H (APC control) and connect the plot to the R3 gated cell population. Import the Tie2 stained sample data on a SSC‐H vs FL‐5‐H (APC) plot. Copy the quadrant thresholds from the isotype control plot and overlay it on the sample dot plot (Figure 6).
51. In order to review if gated selection in step 48 is appropriate, form a new gate (R4) by square mode in the SSC‐H vs FL‐4‐H (PI) plot of about half the width of R2. Manually, establish gate R5 containing both R1 and R4 in the gate list. Connect the 2 SSC‐H vs FL‐5‐H (APC control) and SSC‐H vs FL‐5‐H (APC Tie2) plots to R5 instead of R3. Readjust the location of the threshold within the R5 box, so that the quadrant threshold set in the isotype control SSC‐H vs FL‐5‐H (APC control) dot plot can be shifted to the lowest APC intensity level and highest Tie2 associated intensity (Figure 6).

*Critical*: Adjust gate R4 multiple times to determine the optimal condition. For low cell numbers, this can also include expanding the gate.

52. Adjust the R2 gate in SSC‐H vs PI and shift the quadrant threshold set in the SSC‐H vs FL‐5‐H (APC control) dot plot to the lowest APC fluorescent intensity possible, while maintaining >99% positivity in the left bottom quadrant. When appropriated, copy the quadrant threshold and replace the thresholds set in the sample SSC‐H vs FL‐5‐H (APC Tie2) plot. (Figure 6; See Table 3 for troubleshooting solutions.)
53. The expression level of Tie2 can be obtained from the SSC‐H vs FL‐5‐H (APC Tie2) plot R3 gated cell numbers or as a percentage by summing up the percentages given in the top and bottom right quadrants (Figure 6).

#### 2.3.6 Sorting of Tie2^+^ cells—Timing 3 hours

54. Set up the FACS equipment according to manufacturer instruction.

*Critical*: The below steps describe the methods used for FACS Vantage SE. Consider that the settings and procedures might have to be adjusted for different FACS machinery and associated software.

*Critical*: Considering the cost and time consumption of the FACS procedures, we advise preforming FCM analysis to determine the optimal settings, conditions, and frequencies of the cell populations.

55. Stabilize the FACS equipment for 30 minutes after turning it on (including main‐power, power supply to the lasers, vacuum pomp, and PC) and then set the sheath fluid pressure to 10 PSI.

*Critical*: Nozzle diameter is an important factor for successful NPC collection: 70 μm nozzle diameter results in optimal findings, with decreasing accuracy with increase in nozzle diameter.

56. Adjust the frequency of the nozzle to form a stream of independent droplets. For example, a 21 000/s nozzle frequency is available for the FACS Vantage SE.
57. Apply approximately 4 mL of FACS buffer on the mesh of the cell strainer cap tubes. Discard the filtrate.
58. Stained NPC suspensions obtained during step 37 should individually be transferred to the cap of a 35‐μm mesh cell strainer tube under aseptic conditions. Carefully, wash the mesh with 4 mL of FACS buffer. Pipette lingering solution on the filter mesh repeatedly until all liquid has been filtrated. Formation of bubbles is not of concern.

*Critical*: Ensure the tip of the pipet touches but not deforms the filter to apply the cell suspension directly onto the mesh.

59. Apply a nonfilter cap to the tubes and centrifuge the filtered samples for 5 minutes at 500*g* at 4°C and discard the supernatant.
60. Resuspend the collected cell pellet in 4 mL FACS buffer and reapply the suspension to the cap cell strainer. Pipette lingering solution on the filter mesh repeatedly until all liquid has been filtrated. Formation of bubbles is not of concern.
61. Replace the cap with a sterile nonfilter cap and centrifuge the filtered samples for 5 minutes at 500*g* at 4°C and discard the supernatant.
62. Add 500 μL FACS buffer to each sample to remove static energy and prevent drying out of the samples. Until further usage, keep samples on ice blocked from light.
63. Set up the laser and filter pathways. Adjust the Band Pass Filters; forFL‐1 (FITC labels); Band Pass Filter 530/30, For FL‐2 (PE labels); Band Pass Filter 575/26, For FL‐5 (APC labels); Band Pass Filter 660/20, For SSC‐W and PI; Band Pass Filter 610/20.

*Critical*: Band Pass Filter of PI detection channel recommends 610/20 in order to increase detection sensitivity and resolution of PI.

64. Disinfect the flow path by placing a polystyrene tube filled with 70% ethanol at the flow port, and allow running for 10 minutes. Follow up by a 10‐minute flow of sterile distilled water to wash and dilute reminiscent ethanol in the flow path.
65. Start the analysis using isotype control samples (Tube #1 from Table 1) to set up the gating for SSC‐H and FSC‐H, as well as PI positivity following step 39 to 40. Also establish the threshold for Tie2 positivity by setting the threshold of APC‐intensity to the lowest intensity possible that allows for a population < 1.0% on the right side of the threshold. (See Table 3 for troubleshooting solutions.).

*Critical*: In order to limit aggregation of the suspended NPC, ensure that the density of NPC is within the 1.0 × 10^5^ and 5.0 × 10^5^ cell/mL range. Increase in cell density may lead to aggregation of NPC, which increase the risk of clogging the flow path and nozzle of the machinery.

*Critical*: If the number of NPCs is low, consider following step 61 and resuspend the NPC in a smaller amount of FACS buffer, to enhance the detection rate. Do not increase the flow rate, as this limits cell survivability.

66. Quickly confirm that Tie2 stained samples (Tube #3 from Table 1) fit within the gates established in step 61.
67. Start the sorting procedure at a speed of about 300 to 500 cells/s and collect the Tie2^+^ NPC and Tie2^−^ cells separately in 500 μL FACS buffer supplemented sterile containers. (See Table 3 for troubleshooting solutions.)
68. After completion of the cell sorting procedure, perform general flow path management according to manufacturer instructions to clean and shut down the system.
69. Follow step 70 to 80 to initiate colony‐forming assays, step 21 to 27 for cell expansion, or apply the cells directly to other planned experiments.

*Critical*: Prevent freezing the cells directly after the sorting procedure, as cell survival and Tie2^+^ expression will be severely compromised.

#### 2.3.7 Colony‐forming assay—Timing 7 to 10 days

70. Aliquot or dilute the sorted Tie2^+^ NPC and Tie2^−^ NPC suspension to 4 × 10^3^ cells per condition in 50 μL αMEM.
71. Add 4 mL of MethoCult H4230 methylcellulose medium to a 5‐mL polystyrene FACS tube.
72. Resuspend the cell suspension in methylcellulose medium and vigorously mix by inversion.

*Critical*: During the mixing, limit air bubble formation and avoid vortexing the cell suspension.

73. Gently draw 1 mL of the methylcellulose solution and slowly add this to a 35‐mm petridish.
74. Tilt the plate gently until the solution covers the entire plate.

*Critical*: Prevent any air bubbles from forming.

75. Establish 3 petridishes by repeating step 73 to 74.
76. Add about 2 mL of distilled water to a new 10 cm ø petridish and remove the lid to avoid evaporation of the suspension.
77. Place the methylcellulose‐containing 35 mm ø petridishes from step 75 within the distilled water‐containing 10 cm ø petridish from step 76.

*Critical*: When moving the plates, ensure the water does not flow into the cell‐containing 35 mm plates.

78. Incubate the dishes at 37°C, 5% CO_2_, 5% O_2_ in an incubator for 10 days.

(a) For canine and bovine samples, incubate for 7 days instead of 10 days.

79. Using an inverted light microscope, count the number of spheroid‐shaped colonies (Figure 2) formed within the methylcellulose medium.

*Critical*: Cells adhering to the dish surface should not be counted.

*Critical*: Colonies constituting less than 10 cells should not be counted.

80. Calculate the average number of colonies of 3 methylcellulose 35 mm ø dishes and compare the number of Tie2^+^ and Tie2^−^ colonies.

### Timing

Step 1 to 20 Nucleus pulposus cell isolation: 5 to 10 hours

Step 21 to 27 Expansion of nucleus pulposus cells (optional): 7 days

Step 28 to 37 Antibody staining: 1 to 2 hours

Step 38 to 45 Data collection by FACS: 2 hours

Step 46 to 53 Data analysis and Tie2 detection: 2 hours

Step 54 to 69 Sorting of Tie2^+^ cells: 3 hours

Step 70 to 80 Colony‐forming assay: 7 to 10 days

## 3 RESULTS

Tie2 expression rapidly reduces in NP tissue obtained from patients above the age of 25, while NPs from patients older than 30 years of age demonstrate less than 10% Tie2 expression (Figure 3A). Similarly, Tie2 expression considerably decreases with IVD degeneration, demonstrating an average positivity of approximately 14% for grade III IVDs and less than 2.5% positivity for grade IV IVDs.^8^ As the IVD tissue applied for research purposes is commonly explanted during surgery as part of a treatment of IVD degeneration or scoliosis, or as *postmortem* explantation, it is most likely to be prone to low Tie2^+^ rates. To successfully detect and isolate sufficient Tie2^+^ NPCs from human NP tissue, it is crucial to select appropriate tissue sources and set‐up isolation procedures that minimize the loss of Tie2 expression. Interestingly, the percentage of Tie2^+^ cells differs considerably between fetal human donors (Figure S1), and is generally lower than in young adult individuals. FACS analysis showed only 0% to 17% Tie2 positivity for 22 to 24‐week‐old human fetuses, but these results might have been influenced by cell multiplication, since NPCs of fetal/stillborn individuals need to be expanded to yield >10^3^ cells for FCM or FACS analysis, negatively affecting Tie2 expression. Additionally, it was established that the number of washing steps significantly reduced Tie2 positivity in these specific fetal samples (data not shown). We hypothesize that the mechanical force generated during the washing step with excessive FACS buffer separates the antibody from its antigen, suggesting a weak affinity to the fetal Tie2 membrane receptor.

## 4 DISCUSSION

The percentage of Tie2^+^ cells in stillborn canine NP tissue also varies considerably between donors (Figure S1) and was assessed in the range of 12% to 43% (data not shown), as observed with FACS analysis. Again, these results might have been influenced by cell passaging and cryopreservation, since the NPCs needed to be expanded to obtain sufficient cell numbers for FCM or FACS analysis. Since cell passaging negatively affects Tie2 positivity, the percentage of Tie2^+^ NPCs might have been higher in freshly isolated stillborn canine NPCs. Similar to humans, Tie2 positivity rapidly decreases with age in canines, since NP tissue from 1 until 5‐year‐old canine donors contains only 0% to 1% Tie2^+^ NPCs. Therefore, it is crucial to select very young (<1‐year‐old) canine donors for Tie2‐related research purposes.

In bovine NP tissue, the number of obtained Tie2^+^ cells after digestion of the NP tissue constitutes approximately 5% to 12% from the entire NPC population. No important variation in the number of Tie2^+^ NPCs after isolation was detected, since the bovine coccygeal discs were homogenous and obtained from 8 to 12 months old animals from the local abattoir. It should be noted that only healthy discs were harvested with no sign of degeneration or trauma.

Murine samples also demonstrate an age‐related Tie2^+^ ratio with a relatively low expression observed from 6 months of age.^11^ Additionally, for murine samples in particular, primary NPCs show low Tie2^+^ ratio.

### Author contributions

D.S., M.A.T., and B.G. conceived the studies and initiated the collaboration. D.S. provided funding, data, and drafted the manuscript. J.S. drafted and formatted the manuscript and figures, and provided practical assistance. F.C.B. provided canine and human fetal samples and data, formatted the figures and drafted the manuscript. A.T. provided bovine samples and data, and drafted the manuscript. N.S. provided human samples, and provided human and canine data. Moreover, N.S. drafted the manuscript. Y.N. provided human, canine, and bovine FCM, CFA, and FACS data. S.C.W.C. established the bovine FACS procedures and reviewed the manuscript. T.N. provided human and murine samples and data. L.B.C. provided human fetal samples. D.A.F. provided bovine FACS data. R.M. provided data and assisted in drafting the manuscript. S.G. and M.W. provided funding and assisted in drafting of the manuscript. M.A.T. provided funding and canine and human fetal samples, and was involved in drafting the manuscript. B.G. provided funding, bovine samples, bovine data, and was involved in drafting the manuscript. All authors have read, reviewed, and approved the final version of the manuscript.

### Financial disclosure

The authors would like to acknowledge the Tokai Support center and Tokai animal center for their support in data assessment and animal care. Moreover, we would like to acknowledge the FACSlab core facility of the University of Bern. The authors would also like to thank Frank Riemers (Utrecht University) for help with designing the phylogenetic tree. The study was supported by funding to BG by Hansjörg Wyss and Hansjörg Wyss Medical, United States, and by project‐based funding from the Swiss National Science Foundation project #310030_153411. This work was further supported by funding to MAT by the AOSpine International (SRN2011_11) and the Dutch Arthritis Foundation (LLP22 and LLP12). Furthermore, the AO Spine Research Network supported this work with Exchange Awards to S.G., M.A.T., and D.S.

### Conflict of interest

The authors declare no financial conflicts.

## Supporting information

Figure S1: **Immunohistochemistry for Tie2 in young human and canine donors:** Tie2 immunopositivity varies considerably in nucleus pulposus tissue from young human (20 weeks of pregnancy – 3 months postnatal, *n* = 14) and canine (stillborn, *n* = 11) donors. The upper picture represents the nucleus pulposus donor with the highest number of Tie2 immunopositive cells, while the lower picture represents a nucleus pulposus donor with the lowest number of Tie2 immunopositive cells per species. (Positive) cell numbers were manually counted in each NP using Photoshop CC and the percentage of Tie2 positive cells was calculated per donor.

Figure S2: **Multilineage differentiation potency of bovine Tie 2+ cells.** The differentiation assays were performed in Tie2‐, Tie2+ (i.e. NPPC) cells after sorting. Left column represents the adipogenic differentiation: Oil red O staining showing the formation of fat droplets. Middle column represents the chondrogenic differentiation: safranin‐O staining showing production of proteoglycans. Right column shows the microscopic images of osteogenesis: alizarin red staining showing calcium deposition.
