# Supplementary figures and images for: Successful fishing for nucleus pulposus progenitor cells of the intervertebral disc across species
Source: JOR Spine. 2018 Jun 27;1(2):e1018. doi: 10.1002/jsp2.1018 (PMC6686801; doi:10.1002/jsp2.1018)

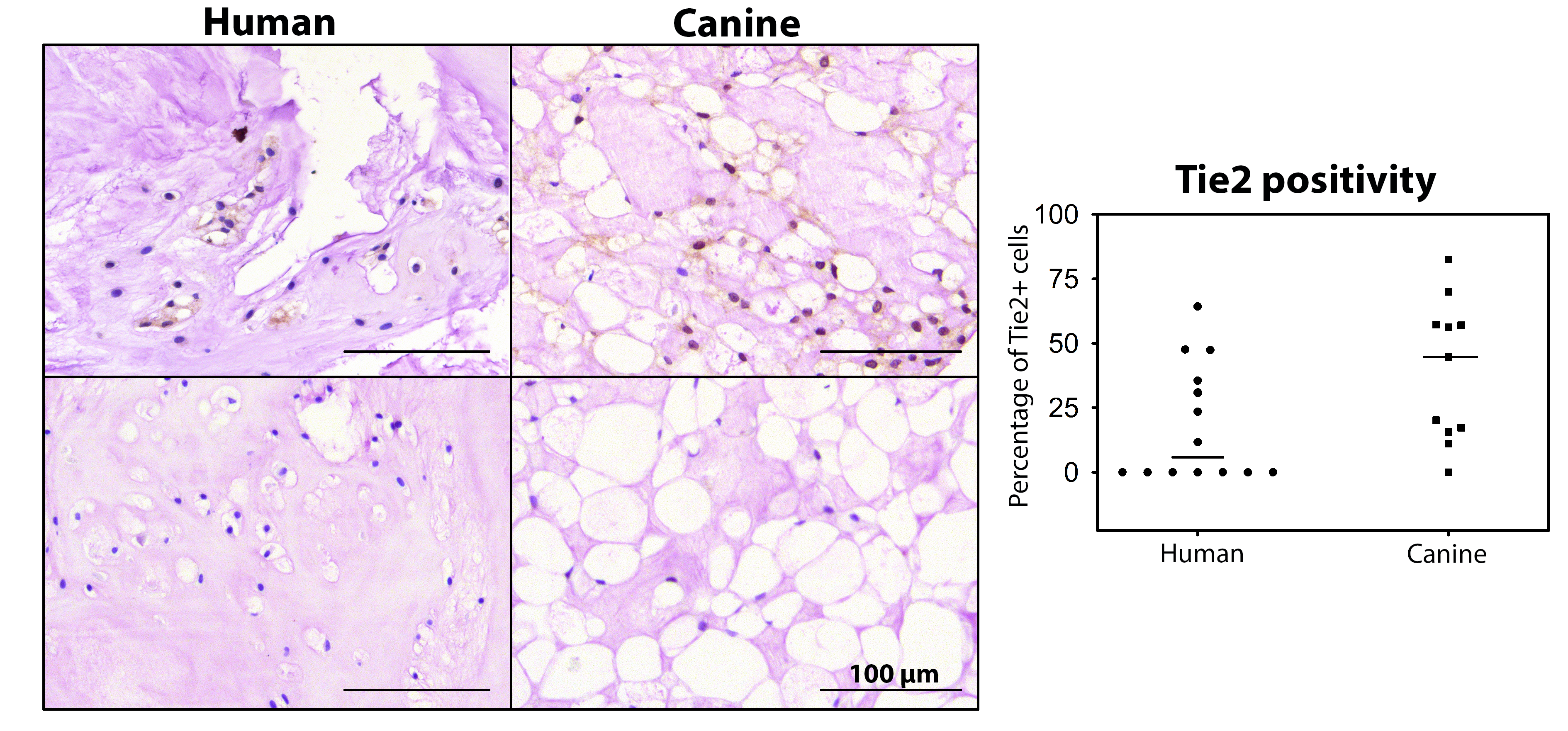

Supplement: Supplementary file 1 — Figure S1: Immunohistochemistry for Tie2 in young human and canine donors: Tie2 immunopositivity varies considerably in nucleus pulposus tissue from young human (20 weeks of pregnancy – 3 months postnatal, n = 14) and canine (stillborn, n = 11) donors. The upper picture represents the nucleus pulposus donor with the highest number of Tie2 immunopositive cells, while the lower picture represents a nucleus pulposus donor with the lowest number of Tie2 immunopositive cells per species. (Positive) cell numbers were manually counted in each NP using Photoshop CC and the percentage of Tie2 positive cells was calculated per donor. [file JSP2-1-e1018-s001.tif]

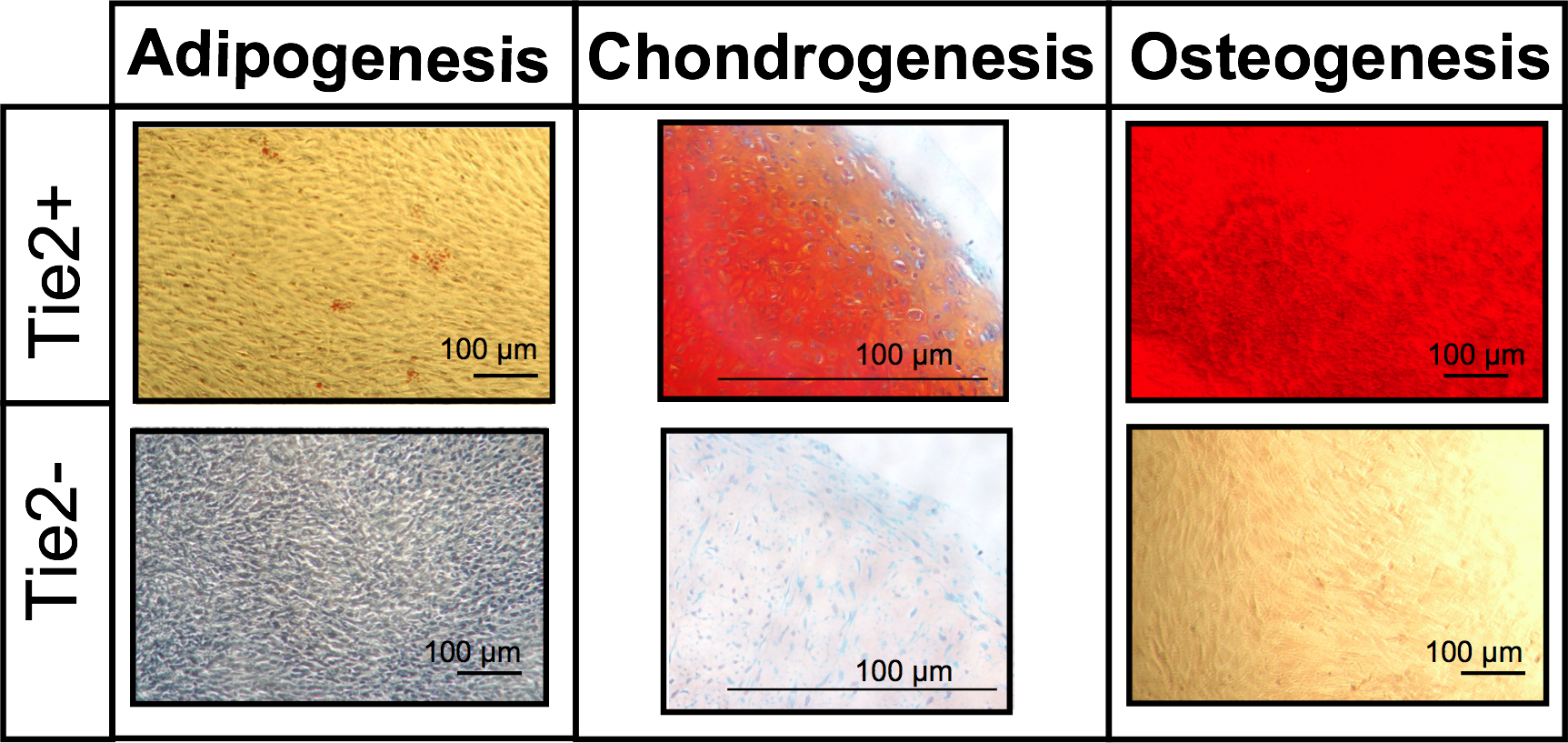

Supplement: Supplementary file 2 — Figure S2: Multilineage differentiation potency of bovine Tie 2+ cells. The differentiation assays were performed in Tie2‐, Tie2+ (i.e. NPPC) cells after sorting. Left column represents the adipogenic differentiation: Oil red O staining showing the formation of fat droplets. Middle column represents the chondrogenic differentiation: safranin‐O staining showing production of proteoglycans. Right column shows the microscopic images of osteogenesis: alizarin red staining showing calcium deposition. [file JSP2-1-e1018-s002.tiff]
